# Sclonal architectures predict clinical outcome in colon adenocarcinoma

**DOI:** 10.1111/jcmm.16208

**Published:** 2020-12-24

**Authors:** Ji Lv, Lili Yan, Yang Lu, Dongfeng Liu, Jia Niu, Liyong Yin

**Affiliations:** ^1^ Department of surgery The First Hospital of Qinhuangdao Qinhuangdao China; ^2^ Department of Neurology The First Hospital of Qinhuangdao Qinhuangdao China

**Keywords:** immunity, tumour evolution, tumour heterogeneity, tumour microenvironment

Colorectal carcinoma (CRC) is among the most common cancers with high morbidity and mortality among all malignancies, and the most common CRC is colon adenocarcinoma (CA).^1^ Although radical resection combined with chemotherapy improved survival,^2^ the 5‐year overall survival rate did not exceed 65%.^3^, ^4^, ^5^ To understand the aetiology of CRC, a large number of studies have been conducted to screen genetic changes in patients.^6^, ^7^, ^8^, ^9^ The genetic landscape of CRC is characterized by increased mutations, carcinogenic WNT pathway changes, microsatellite instability, copy number amplification and recurrent chromosomal translocations, including APC, TP53, SMAD4, PIK3CA and KRAS, ARID1A, SOX9 and FAM123B/WTX mutations and potential drug‐targeted amplification of ERBB2 and copy number amplification of IGF2 (Figure 1A‐F). Comprehensive analysis of genetic and clinical information suggests that certain somatic copy number variation (SCNA) or mutant driving genes may be potential prognostic markers.^10^ The development of cancer is driven by the gradual accumulation of somatic changes, and mutations obtained at different stages of tumour development may be associated with different clinical outcomes.^11^ However, the chronological sequence of somatic events and their potential clinical effects during CRC evolution have not been fully studied. The evolutionary pattern of CRC has great patient heterogeneity. Among them, TP53, KRAS and APC are considered to be the most common earliest events, which may be used as CRC driver events. CSMD3, TTN and ERBB4 appear at the latest in CRC and may be related to the progression of CRC (Figure 1). Tumours with a high mutation load are more likely to respond to checkpoint blockade strategies against immunosuppression.^12^, ^13^, ^14^, ^15^ Survival analysis showed that the clonal events of the early gene APC had a greater impact on the prognosis than the sub‐clonal events, and late ERBB4 clonal/sub‐clonal events had a poor prognosis for total survival (Figure 2). For a few types of tumours, more convincing evidence has been established that multiple sub‐clones have genetic changes in the same gene or in genes that play a role in the same pathway.^16^, ^17^ Studies provide evidence that Darwinian evolution effect for at least part of the ITH: one or more sub‐cloning‐driven events in different sub‐clones of the tumour.^16^, ^18^, ^19^, ^20^, ^21^, ^22^ There was a significant difference in the amount of N and Stage staging in clonal events and a significant difference in clonal events in patients with recurrence events (Figure 2). There is also a significant positive correlation between clonal events and tumour mutation load (TMB) and new antigen production. The clonal events of samples from the MMR mutant group were significantly higher than those from the non‐mutant group, which highlighted the importance of systematic assessment of evolutionary patterns in CRC clinical management (Figure 2).

**FIGURE 1:**
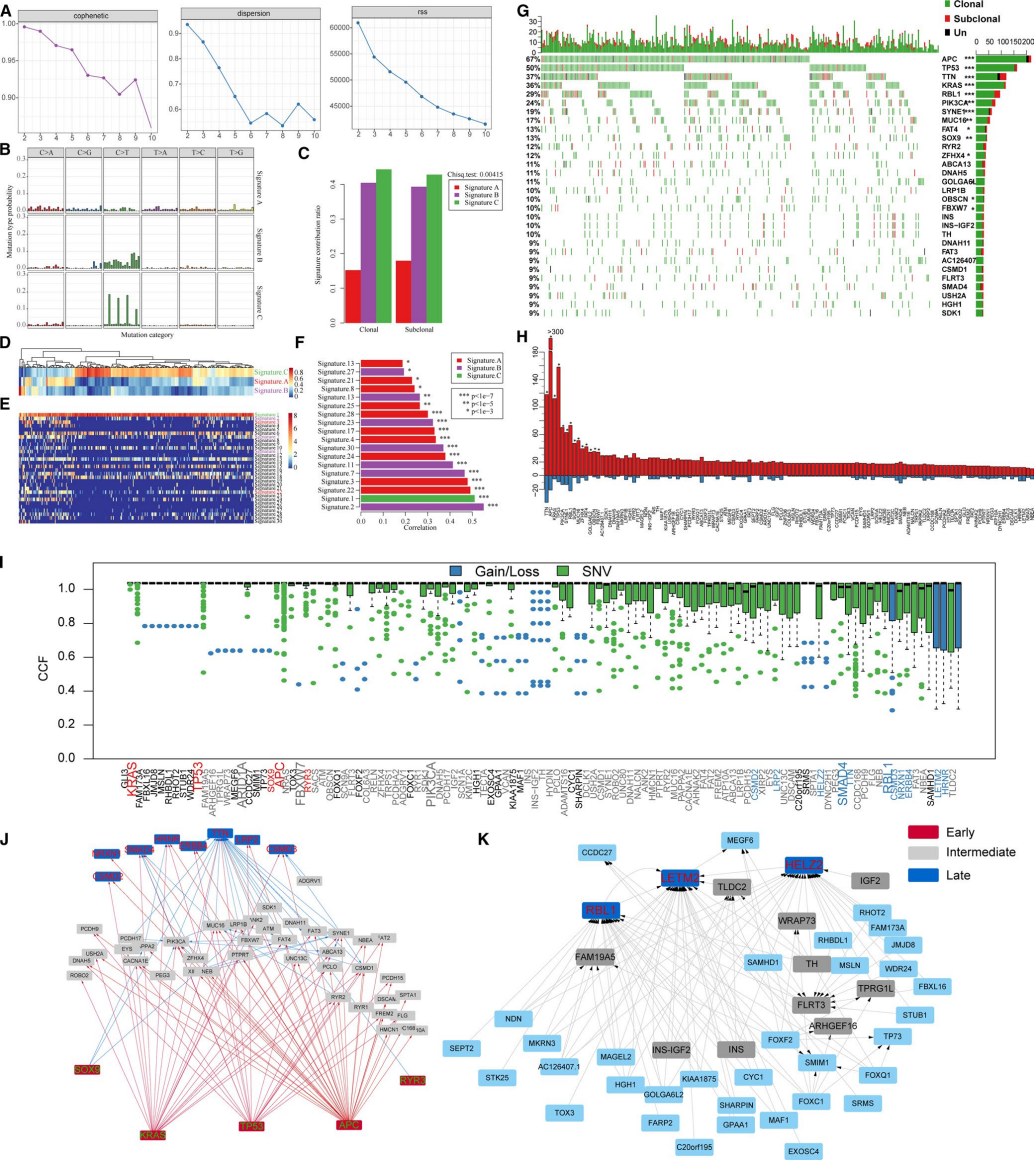
Mutation signature distribution of The Cancer Genome Atlas (TCGA) Colon cancer (COAD) samples. A, Cophenetic, dispersion and RSS distribution when rank = 2‐10. B, 3 single nucleotide variant (SNV) mutation signatures identified by consensus non‐negative matrix factorization (NMF). C, Comparison of clonal and sub‐clonal mutations and signatures. D, 3 signatures of Colon cancer (COAD) samples. E, Composition of 30 signatures of Colon cancer (COAD) samples provided by Catalog of Somatic Mutations in Cancer (COSMIC). F, The similarity between signature A‐C and signature 1‐30. G, Distribution of clonal and sub‐clonal events in CRC samples (Top 30 genes). Top panel: Statistics on the number of clonal and sub‐clonal. Bottom panel: The clonal and sub‐clonal status of the CRC sample. Genes marked with '*' indicate genes with significant differences in clonal/sub‐clonal events. Un indicates that it cannot be determined as clonal or sub‐clonal events. H, Clonal and sub‐clonal enrichment results of mutation rate of > 5% gene (SNV + CNV). * indicates significant enrichment (*P* < .05). I, Cancer cell fraction (CCF) distribution of top 5% mutant genes. Genes that only contains the clone mutation were removed. J, The temporal maps of SSNV acquisitions in Colon cancer (COAD). K, The temporal maps of somatic copy number variation (SCNV) acquisitions in Colon cancer (COAD). The arrow indicates that two genes appear in the same sample. The width of the arrow indicates the number of times the event occurred. Red represents the genes that appear early, grey represents the genes that appear in the intermediate phase, blue represents genes that appear late, and other colours indicate that the temporal genes cannot be determined. The temporal order significance test was false discovery rate (FDR) < 0.05

**FIGURE 2:**
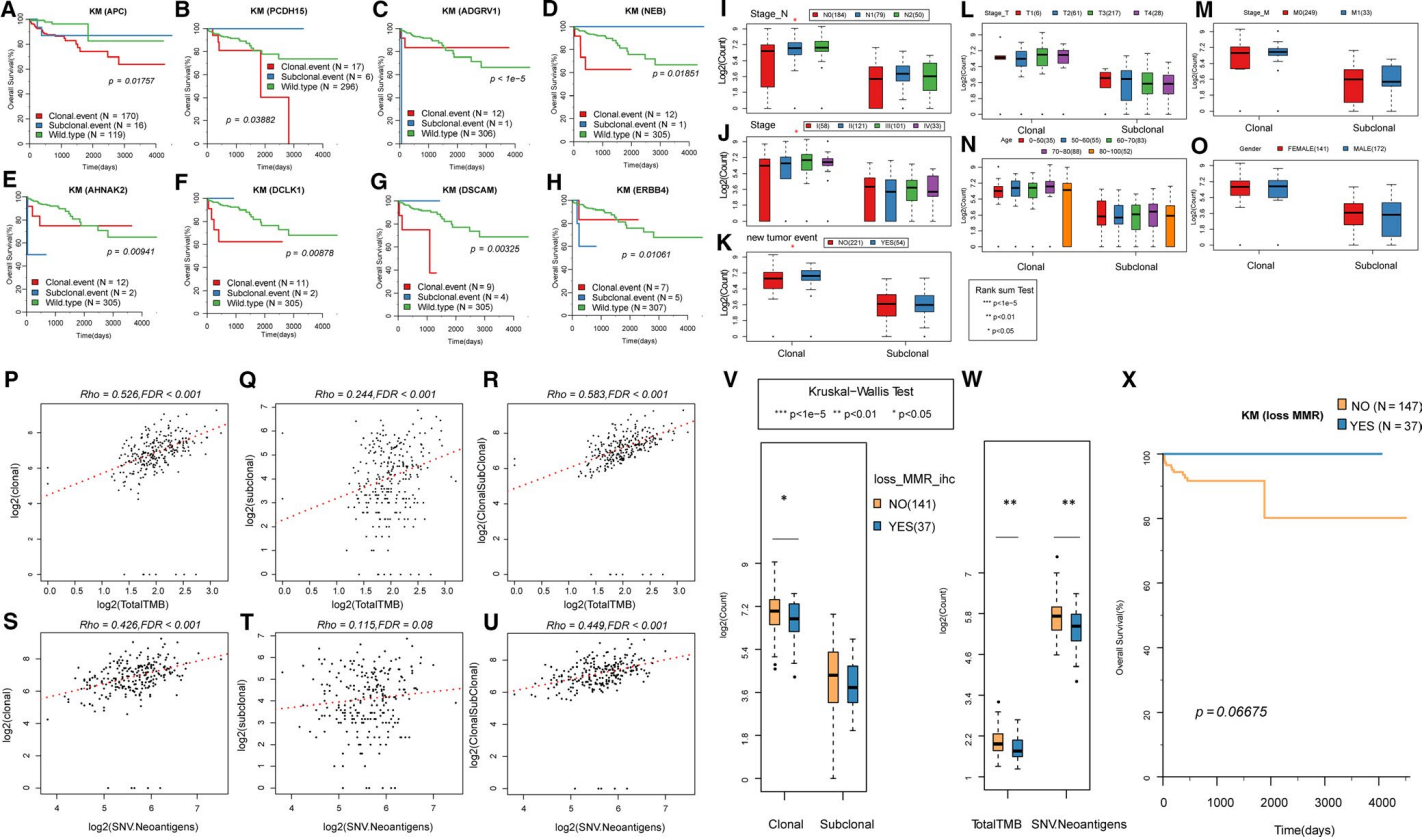
The relationship between overall survival and the clonal/sub‐clonal status of somatic copy number variation (SCNV) and single nucleotide variant (SNV). A, Kaplan‐Meier (KM) curve of clonal/sub‐clonal state of early genes and overall survival (OS). B‐G, Kaplan‐Meier (KM) curve of clonal/sub‐clonal state of metaphase genes and overall survival (OS). H, Kaplan‐Meier (KM) curve of late gene clonal/sub‐clonal state and overall survival (OS). P values are calculated by log rank sum test. I, The number distribution of Clonal events and sub‐clonal events in the N staging. J, The number distribution of Clonal events and sub‐clonal events in the Stage staging; K, the number distribution of Clonal events and sub‐clonal events on the recurring (YES) and non‐recurrent (NO) groups. L, The number distribution of Clonal events and sub‐clonal events in the T staging. M, The number distribution of Clonal events and sub‐clonal events in the M staging. N, The number distribution of Clonal events and sub‐clonal events in the Age. O, The number distribution of Clonal events and sub‐clonal events in the gender. P, Scatter plot of clonal events and TMB. Q, Scatter plot of sub‐clonal events and TMB. R, Scatter plot of clonal + sub‐clonal events and TMB. S, Scatter plot of clonal events and neoantigens. T, Scatter plot of sub‐clonal events and neoantigens. U, Scatter plot of clonal + sub‐clonal events and neoantigens. V, Comparison of clonal/sub‐clonal events between MMR defect group (YES) and normal group (NO). W, Comparison of neoantigens between MMR defect group (YES) and normal group (NO). X, Comparison of overall survival (OS) between MMR defect group (YES) and normal group (NO). Spearman method (Rho value) was used for correlation test. The MMR status comes from the IHC results

Overall, we obtained genome‐wide sequencing data from a cancer genome atlas of 319 patient cohorts (Table 1). We inferred the chronology, mutation characteristics and evolutionary patterns of frequent somatic events in CRC and evaluated their clinical relevance in CRC patients. Our findings revealed the mutation characteristics and ITH changes in CRC evolution, proposed a presumptive evolutionary model of CRC development and identified some clonal or sub‐clonal events as potential prognostic markers.

**TABLE 1:** Clinical informations of pre‐treated COAD data set

Clinical	No. of samples
OS_Event	
Alive	285
Dead	34
Stage_T	
T1	7
T2	61
T3	222
T4	28
Unknown	1
Stage_N	
N0	189
N1	80
N2	50
Stage_M	
M0	250
M1	33
Unknown	36
Stage	
I	58
II	122
III	101
IV	33
Unknown	5
Gender	
FEMALE	144
MALE	175
Age	
0‐50	36
50‐60	57
60‐70	84
70‐80	90
80‐100	52
Histological type	
Colon Adenocarcinoma	286
Colon Mucinous Adenocarcinoma	31
Clonal/Sub‐clonal count	
Median clonal	101
Median sub‐clonal	11

## AUTHOR CONTRIBUTION

**Ji Lv:** Conceptualization (equal). **Lili Yan:** Data curation (equal). **Yang Lu:** Software (equal). **Dongfeng Liu:** Methodology (equal). **Jia Niu:** Writing‐original draft (equal). **Liyong Yin:** Writing‐review & editing (equal).
